# Variability of polygenic prediction for body mass index in Africa

**DOI:** 10.1186/s13073-024-01348-x

**Published:** 2024-05-30

**Authors:** Tinashe Chikowore, Kristi Läll, Lisa K. Micklesfield, Zane Lombard, Julia H. Goedecke, Segun Fatumo, Shane A. Norris, Reedik Magi, Michele Ramsay, Paul W. Franks, Guillaume Pare, Andrew P. Morris

**Affiliations:** 1 SAMRC/Wits Developmental Pathways for Health Research Unit, Department of Pediatrics, Faculty of Health Sciences, University of the Witwatersrand, Johannesburg, South Africa; 2 Sydney Brenner Institute for Molecular Bioscience, Faculty of Health Sciences, University of the Witwatersrand, Johannesburg, South Africa; 3 Harvard Medical School, Boston, MA USA; 4 Channing Division of Network Medicine, Brigham and Women’s Hospital, 181 Longwood Avenue, Boston, MA 02115 USA; 5 Estonian Genome Centre, Institute of Genomics, University of Tartu, Tartu, Estonia; 6 Division of Human Genetics, National Health Laboratory Service, and School of Pathology, Faculty of Health Sciences, University of the Witwatersrand, Johannesburg, South Africa; 7 Biomedical Research and Innovation Platform, South African Medical Research Council, Cape Town, South Africa; 8 NCD Genomics, MRC/UVRI LSHTM Uganda Research Unit, Entebbe, Uganda; 9 Precision Healthcare University Research Institute (PHURI), Queen Mary University of London, London, UK; 10 School of Human Development and Health, University of Southampton, Southampton, UK; 11 Department of Clinical Sciences, Lund University, Helsingborg, Sweden; 12 Department of Nutrition, Harvard T. H. Chan School of Public Health, Boston, MA USA; 13 Department of Pathology and Molecular Medicine, McMaster University, Hamilton, Canada; 14 Centre for Genetics and Genomics Versus Arthritis, Centre for Musculoskeletal Research, University of Manchester, Manchester, UK

**Keywords:** BMI, Variability, Polygenic prediction, Polygenic risk score

## Abstract

**Background:**

Polygenic prediction studies in continental Africans are scarce. Africa’s genetic and environmental diversity pose a challenge that limits the generalizability of polygenic risk scores (PRS) for body mass index (BMI) within the continent. Studies to understand the factors that affect PRS variability within Africa are required.

**Methods:**

Using the first multi-ancestry genome-wide association study (GWAS) meta-analysis for BMI involving continental Africans, we derived a multi-ancestry PRS and compared its performance to a European ancestry-specific PRS in continental Africans (AWI-Gen study) and a European cohort (Estonian Biobank). We then evaluated the factors affecting the performance of the PRS in Africans which included fine-mapping resolution, allele frequencies, linkage disequilibrium patterns, and PRS-environment interactions.

**Results:**

Polygenic prediction of BMI in continental Africans is poor compared to that in European ancestry individuals. However, we show that the multi-ancestry PRS is more predictive than the European ancestry-specific PRS due to its improved fine-mapping resolution. We noted regional variation in polygenic prediction across Africa’s East, South, and West regions, which was driven by a complex interplay of the PRS with environmental factors, such as physical activity, smoking, alcohol intake, and socioeconomic status.

**Conclusions:**

Our findings highlight the role of gene-environment interactions in PRS prediction variability in Africa. PRS methods that correct for these interactions, coupled with the increased representation of Africans in GWAS, may improve PRS prediction in Africa.

**Supplementary Information:**

The online version contains supplementary material available at 10.1186/s13073-024-01348-x.

## Background

Obesity is increasing globally. In 2020, one-third of the global population was estimated to be overweight or obese [1]. It is predicted that, by 2030, if drastic measures are not taken to curtail this burden, the prevalence of obesity will reach 50% [1]. African populations have not been spared: they account for 77% of low-middle-income countries that carry 80% of the obesity burden [2]. Evidence from twin studies suggests that body mass index (BMI) heritability is 40–70% [3] and genome-wide association studies (GWAS) have identified hundreds of contributing loci [4]. However, the largest GWAS of BMI have predominantly been undertaken in populations of European and East Asian ancestry [4–6]. More recent, smaller-scale GWAS undertaken in other ancestry groups, including the African Partnership of Chronic Disease Research network in continental Africa, have not yielded additional novel BMI loci, presumably due to low power [7, 8]. Multi-ancestry meta-analysis of BMI GWAS enhances the discovery of loci contributing to obesity across populations and improves the opportunities for localizing the causal variants driving association signals at these loci by taking advantage of the differences in the structure of linkage disequilibrium (LD) between diverse ancestry groups [9, 10]

The underrepresentation of continental Africans in global genomic studies of complex traits increases the risk of Africa being left behind in genomic-driven precision medicine efforts, further worsening global health disparities [11]. Polygenic risk scores (PRS) enhance risk stratification, essential for precision medicine efforts. However, the transferability of PRS derived from European ancestry GWAS to other ancestry groups, including Africans and African Americans, is often poor owing to differences in allele frequencies, LD structure, and environmental factors [11, 12]. Other multi-ancestry studies, such as the Million Veteran Program and the All of Us initiative, are now increasing the representation of African Americans in GWAS [13]. However, due to important genetic differences between continental Africans and admixed African Americans [14], greater representation of the former in genetic studies is necessary to enhance prediction.

Little is known about the factors contributing to BMI PRS prediction variability in Africa. Although genetic diversity has been noted as a contributing factor through simulation studies in Uganda, it is unclear how differences in allele frequencies, LD patterns, and gene-environment factors affect the portability of the PRS within other West, East, and South regions of Africa [15]. In view of resource limitations in Africa, understanding these parameters might help in developing tools that improve the generalizability of PRS in Africa, thereby enhancing its utilization in future precision medicine efforts. Therefore, in this study, we have assembled previously published GWAS of BMI across multiple ancestry groups to compute a multi-ancestry PRS that was used to assess the factors that affect the generalizability of polygenic prediction of BMI in continental Africans.

## Methods

### Cohorts and Biobanks used for the BMI GWAS

We assembled GWAS of BMI across diverse ancestry groups that were imputed to the 1000 Genomes Project or Haplotype Reference Consortium reference panels from the UK Biobank (UKBB), Biobank Japan (BBJ), the African Partnership for Chronic Disease Research (APCDR), Network and the Population Architecture and Genetic Epidemiology (PAGE) study. BMI was inverse rank normalized in all the studies considered for the meta-analysis. These discovery studies and the two target data sets AWIGen and EstBB are briefly described below.

Full details of BMI GWAS analyses in the UKBB have been previously reported [16]. The UKBB is a large-scale biomedical database comprised of half a million UK participants with de-identified genetic and health information. For our study, we considered 456,422 individuals of European ancestry. Imputation was performed using the 1000 Genomes Project (Phase 3) reference panel, resulting in 8,531,416 variants after excluding those with minor allele frequency (MAF) < 0.01 and missingness of > 0.1. Genetic association analysis was undertaken using FastGWAS in which linear mixed models were fitted for inverse rank normalized BMI residuals while adjusting for age, age^2^, and sex. The random effect for the genetic relationship was included to account for population structure and relatedness. These summary statistics are accessible through this link (https://yanglab.westlake.edu.cn/data/ukb_fastgwa/imp/pheno/21001) [16].

The Biobank Japan (BBJ) is a prospective genome biobank that recruited participants from 12 medical institutions in Japan. BBJ GWAS of BMI comprised 158,284 individuals of East Asian ancestry [6]. Imputation was conducted using East Asian populations in the 1000 Genomes Project (Phase 3) as a reference, and after quality control, there were 6,108,953 SNPs. Residuals fitted for BMI while adjusting for age, age^2^, and sex were transformed using the inverse rank normalization. Linear models were then fitted for the allele dosages while adjusting for the first 10PCs using mach2qtl. Summary statistics were accessed from the Japan Biobank via this link (https://ftp.ebi.ac.uk/pub/databases/gwas/summary_statistics/GCST004001GCST005000/GCST004904/) [6].

The APCDR Network conducted a meta-analysis of GWAS summary statistics from the Uganda, Durban Diabetes Study (DDS), Durban Diabetes Case–Control Study (DDC), and AADM cohorts in 14,126 individuals for multiple traits, including BMI [7]. Imputation was performed using a merged reference panel of the whole-genome sequences from the African Genome Variation Project, Uganda sequences, and the 1000 Genomes Project (Phase 3). An imputation info filtering threshold of 0.3 and a minimum MAF of 0.5% were applied, resulting in 24,423,923 SNPs after quality control. Before meta-analysis, the inverse rank normalized residuals of BMI were fitted in linear mixed models while adjusting for age, age.^2^, and sex. The Han-Eskin random effects meta-analysis approach implemented in METASOFT (RE2) was used to aggregate summary statistics from these four cohorts. The summary statistics are accessible at (https://ftp.ebi.ac.uk/pub/databases/gwas/summary_statistics/GCST009001GCST010000/GCST009057/) [7].

The PAGE study recruited individuals of diverse ancestries who reside in the USA [8]. In this study, 22,216 Hispanics/Latinos, 17,299 African Americans, 4680 Asians, 3940 Native Hawaiians, 652 Native Americans, and 1052 individuals of other ancestries, totaling 49,839 participants were enrolled. Imputation was conducted using the 1000 Genomes Project (Phase 3) reference panel, and SNPs with an imputation information score > 0.4 (39,723,562) were included in the analysis. Linear mixed models for the inverse rank normalized residuals for BMI were fitted while adjusting for 10PCs in a joint analysis of all the individuals of varied ancestries. The summary statistics were accessed from the GWAS catalog (https://ftp.ebi.ac.uk/pub/databases/gwas/summary_statistics/GCST008001GCST009000/GCST008025/) [8].

The AWIGen study recruited participants from four African countries that are representative of the East, West, and South regions of Africa [17]. Imputation was performed on the cleaned dataset (with 1,729,661 SNPs and 10,903 individuals, that remained after quality control, which included the removal of closely related individuals) using the Sanger Imputation Server and the African Genome Resources as reference panel. We selected EAGLE2 for pre-phasing and the default PBWT algorithm was used for imputation. After imputation, poorly imputed SNPs with info scores less than 0.6, MAF less than 0.01, and HWE *P*-value less than 0.00001 were excluded. The final QC-ed imputed data had 13.98 M SNPs.

The Estonian Biobank (EstBB) is made up of volunteers resident in Estonia [18, 19]. A total of 136,421 individuals were genotyped using the Illumina Global Screening Arrays (GSAs) and we imputed the dataset to an Estonian reference created from the whole-genome sequence data of 2244 participants Individuals with BMI values 12 > BMI > 65 were removed, quality control, which included the removal of related individuals was performed resulting in 84,578 individuals remaining for analysis. For this analysis, the ESTBB target data set was randomly split into validation (*N* = 8456) and testing (*N* = 76,096) datasets and then utilized in the PRS computation.

### Assessment of lifestyle factors in AWI-Gen

Lifestyle factors were captured using questionnaires in AWI-Gen [17, 19]. Physical Activity was captured using the Global Physical Activity Questionnaire (GPAQ). Smoking status was categorized as never and ever smoked. The sum of household assets was used as a proxy of socioeconomic status. Alcohol use was categorized as; never consumed, current non-problematic consumer, current problematic consumer, or former consumer [20]. Problematic drinkers were defined as those who answered two of the following responses based on the CAGE (cut-annoyed-guilty-eye) questionnaire [21]: Have you ever felt that you should cut down on your drinking? Have people annoyed you by criticizing your drinking? Have you ever felt bad or guilty about your drinking? Have you ever had an alcoholic drink first thing in the morning to steady your nerves, or get rid of a hangover? In the past year, did you ever have 6 or more alcoholic drinks in a single morning, afternoon, or night?

### Multi-ancestry meta-analysis

We aggregated GWAS summary statistics from the UKBB, BBJ, APCDR Network, and PAGE study using the fixed-effects inverse-variance weighted meta-analysis implemented in METASOFT to generate our multi-ancestry meta-analysis discovery dataset. Notably, we applied double genomic control to control for population structure. The square roots of the LDSC intercepts from the UKBB and Japan Biobank were multiplied with the standard errors of the individual studies for single genomic control. In view that PAGE is a multi-ancestry, it was not plausible to obtain an LDSC intercept representative of diversity. An initial run of the meta-analysis was run using the LDSC-corrected summary statistics. Double genomic controls were then implemented using the lambda from this initial analysis in the subsequent meta-analysis to correct for population structure. Overall, our meta-analysis included 678,671 individuals and 21,338,816 biallelic SNPs, each reported in at least two or more studies.

### Associated locus definition in GWAS from UKBB and multi-ancestry meta-analysis

We selected lead SNPs attaining genome-wide significant evidence of association (*p* < 5 × 10^−8^) in the two discovery datasets — (1) UKBB (European only) and (2) multi-ancestry meta-analysis — that were separated by at least 1 Mb. Loci were then defined by the flanking genomic interval mapping 1 Mb up and downstream of lead SNPs.

### Fine mapping

We performed fine-mapping to identify potential causal variants driving BMI association signals for each locus attaining genome-wide significance in the multi-ancestry meta-analysis using a Bayesian approach^23^. The Bayes’ factor (BF) for the *i*th SNP was computed as.

$${BF}_{i}=exp\left[\frac{{Z}_{i}^{2}-log({K}_{i})}{2}\right].$$

In this expression, *K*_*i*_ denotes the number of studies reporting summary statistics for the *i*th SNP, and ${Z}_{i}=\frac{{\beta }_{i}}{\text{SE}({\beta }_{i})}$, where ${\beta }_{i}$ denotes the effect size, and SE (${\beta }_{i})$ is the corresponding standard error for the *i*th SNP. We then calculate the posterior probability, ${\pi }_{i}$, that the *i*th SNP is driving the association signal at a locus by.

$$\pi_i=\frac{{BF}_i}{\sum_{j\;}{BF}_j},$$

where the summation in the denominator is of all SNPs at the locus. The 99% credible set for the locus was computed by ranking all SNPs according to their posterior probability *π*_*i*_ from the highest to the lowest until their cumulative posterior probability reached or exceeded 0.99. We conducted fine-mapping using association summary statistics from the multi-ancestry meta-analysis in the UKBB (European ancestry-specific).

### Polygenic score prediction in AWI-Gen and the Estonian Biobank (EstBB)

**Fig. 1:**
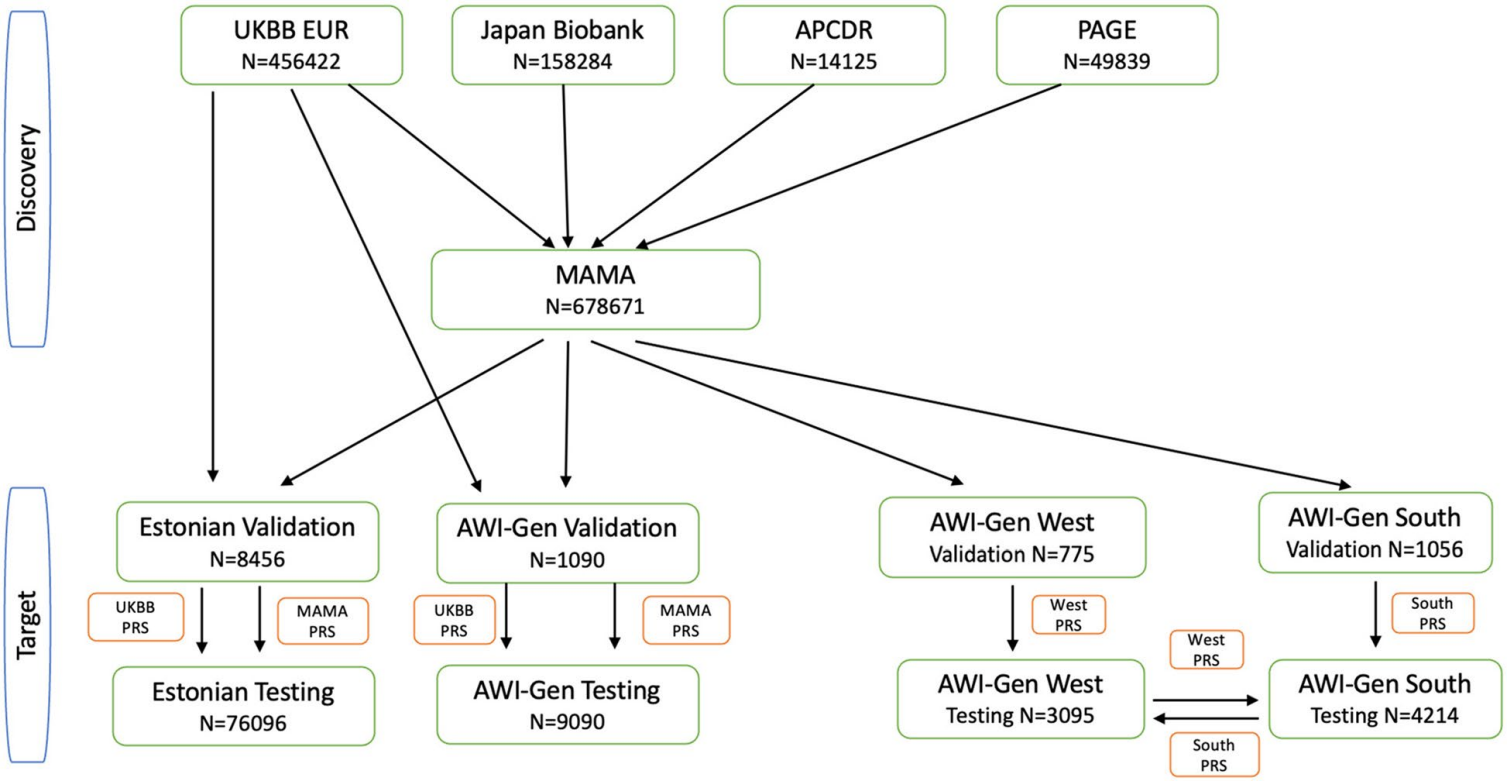
The schematic diagram for the UKBB and MAMA discovery data sets that were used to train the UKBB, MAMA, in the Estonian Biobank and AWI-Gen target data set. The MAMA discovery data was used for the computation of the South and West PRS

The PRSice 2 software implemented the clumping and threshold approach for developing PRS. Summary statistics from the UKBB and multi-ancestry meta-analysis were used as “base” datasets, while AWI-Gen (10,900 participants) genotype data were used as the “target” dataset. The optimal parameters (clumping distance and LD) were determined by computing the PRS in the combined dataset at various combinations of clumping distance and LD (Table S4). This target dataset was randomly split into validation (*N* = 1059) and testing (*N* = 9809) datasets while ensuring representation by sex and regions of Africa (Fig. 1, Table S1). A clumping distance of 250 kb and *r*^2^ of 0.8 where the optimal parameters in AWI-Gen were used to develop the PRS in the testing dataset whilst adjusting for age, sex, and 10 principal components. The best PRS was selected based on the BMI variance explained (see Fig. S1)in the AWI-Gen validation dataset and was used to compute PRS deciles. We used the same procedure to evaluate the performance of the multi-ancestry PRS and UKBB PRS in European ancestry participants from EstBB. The EstBB target data set was randomly split into validation (*N* = 8456) and testing (*N* = 76,096) datasets and similar PRS computation parameters were used as had been done in AWI-Gen. Using the same discovery datasets, we trained a PRS using the PRSCSx with the combinations of APCDR, UKBB, and BBJ. We compared this with a PRS trained without the African population (APCDR). In the PRSCx analysis, we used the ancestry-specific GWAS summary statistics with the 1000G references. We evaluated the best linear combination of the training dataset and then evaluated its predictive in the test dataset.

### Interaction of multi-ancestry PRS with sex and lifestyle variables

Boxplots were constructed to show differences in BMI distribution by sex in the AWI-Gen test dataset. Analysis of variance, stratified by sex, was then performed to compare the mean difference in BMI across the deciles of the multi-ancestry PRS. Linear models were used to test the interaction of the multi-ancestry PRS with physical activity, socioeconomic status, smoking status, and alcohol status while correcting for age, sex, and principal components.

### PRS prediction across regions of continental Africa

We split the target dataset from AWI-Gen according to geographic region: South (*N* = 5270), West (*N* = 3870), and East (*N* = 1760). Boxplots were constructed to illustrate the distribution of BMI in each region. Then the multi-ancestry PRS prediction was evaluated separately in these three data sets using PRSice while adjusting for age, sex, and residual population structure using five principal components. PRS predictivity was indicated as incremental variance (full model with PRS − null model without the PRS). The distribution of physical activity patterns was evaluated across the African regions using boxplots. The interaction of the multi-ancestry PRS and physical activity in the AWI-Gen validation data set was explored using linear models that correct age, sex, and five principal components in the analysis. An interaction plot was computed using the interactions package in R.

### Polygenic prediction of BMI in the West and South regions of Africa

We used the multi-ancestry meta-analysis as a discovery dataset to develop South and West region-specific PRS as they had the largest difference in prediction compared to the East as shown in Fig. 4. The optimal parameters (clumping distance and LD) were determined by computing the PRS separately in the East and South datasets at various combinations of clumping distance and LD (Table S5–S6). We split the South target dataset randomly into validation and testing datasets and then did the same for the West target dataset. A clumping distance of 250 kb and *r*^2^ of 0.8, the optimal parameters (Fig. S1), in AWIGen were used to develop the PRS in each testing dataset whilst adjusting for age, sex, and principal components. The best region-specific PRS was then selected based on BMI incremental variance explained (full model with PRS − null model without the PRS) in the region-matched validation dataset. We also tested the South African-specific PRS in the West African testing dataset and the West African-specific PRS in the South African testing dataset (Table S4–S6). We calculated the Pearson correlation coefficient between South and Western allele frequencies for SNPs in the South and West region-specific PRS. We also plotted LD *r*^2^ against physical distance in West and South Africa for the same SNPs.

## Results

**Fig. 2:**
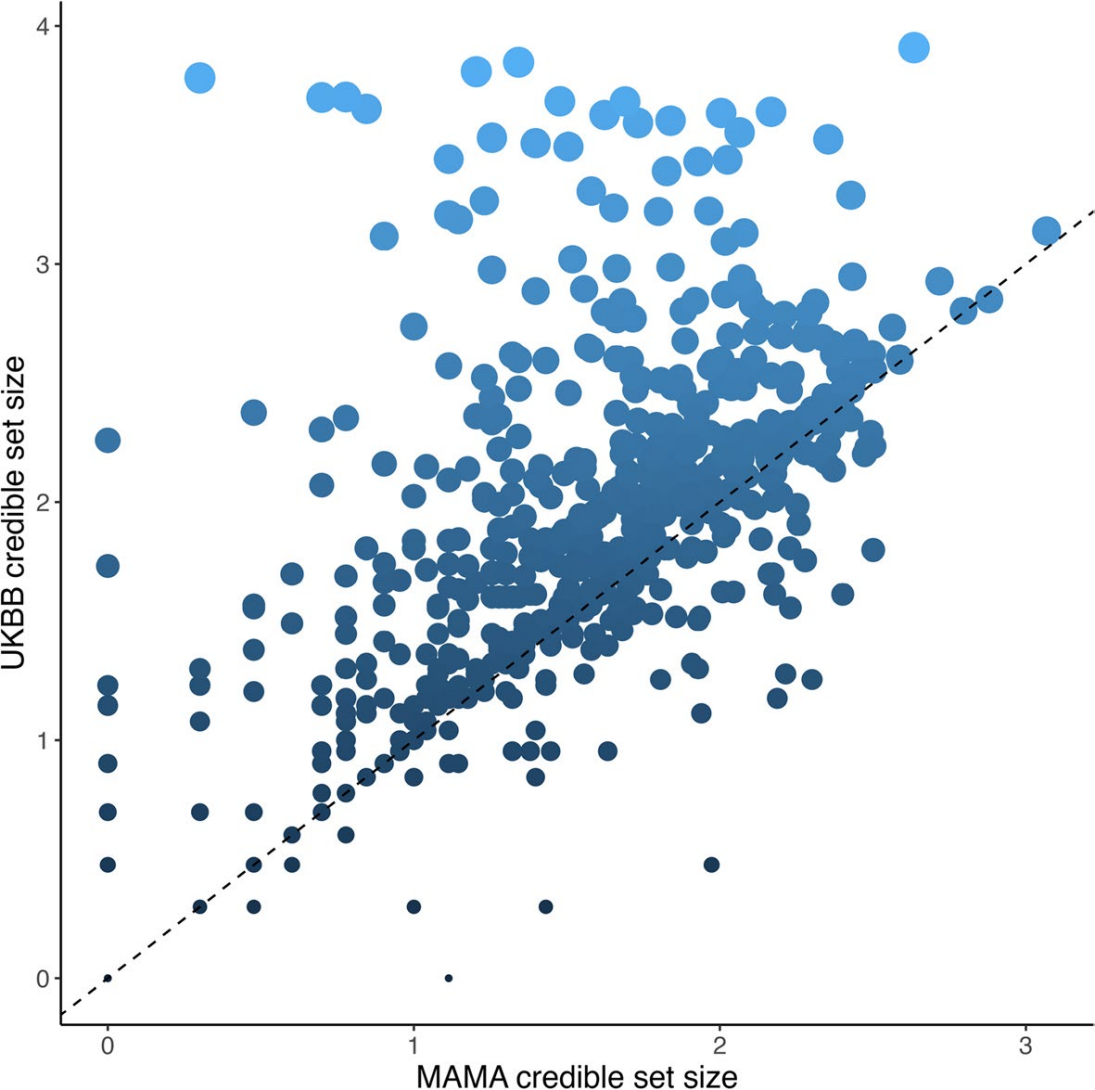
Fine-mapping plot comparing 99% credible set sizes (on log_10_ scale) at loci attaining genome-wide significance (*P* < 5 × 10^−8^) in the UKBB (European ancestry-specific) and multi-ancestry meta-analysis (MAMA). Most loci are above the *y* = *x* line, indicating more refined fine mapping in the multi-ancestry meta-analysis compared to the UKBB

We aggregated previously published GWAS summary statistics for BMI with high-density imputation in 678,545 individuals from multiple ancestry groups through inverse variance weighted, fixed-effects multi-ancestry meta-analysis (Methods). We considered 21,338,816 biallelic single nucleotide polymorphisms (SNPs), each reported in at least two studies. In the multi-ancestry meta-analysis, we identified 5 loci attaining genome-wide significance (*p* < 5 × 10^−8^), defined by lead SNPs separated by at least 1MbWe then assessed whether the localization of putative causal variants driving associations at the 576 BMI loci improved by leveraging differences in the structure of LD between populations contributing to the multi-ancestry meta-analysis. We constructed 99% credible sets of SNPs for each locus based on association summary statistics from the multi-ancestry meta-analysis and the UKBB (Fig. 2). The median 99% credible set size was 58 in the UKBB but just 32 in the multi-ancestry meta-analysis. Furthermore, the 99% credible set was resolved to a single SNP at 16 loci in the multi-ancestry meta-analysis, compared to just nine loci in the UKBB. These results highlight the improved fine-mapping resolution of BMI association signals offered by the diverse ancestry groups contributing to the multi-ancestry meta-analysis.

**Fig. 3:**
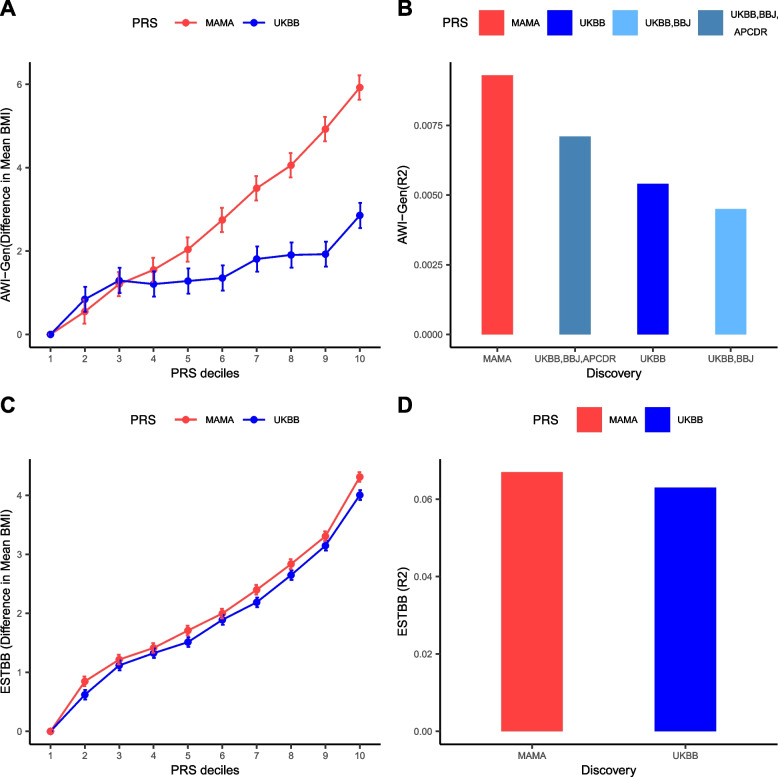
PRS prediction in African and European populations. **A** Difference in mean BMI between PRS deciles (reference is the first decile) in the AWI-Gen target dataset for PRS derived from the MAMA and UKBB. **B** BMI predictions of the following PRSs, MAMA, UKBB, PRSCSx computed from Africans (APCDR), East Asians (BBJ), and Europeans (UKBB)), together with PRSCSx computed from East Asians and Europeans (UKBB, BBJ) in the AWI-Gen target data set. **C** Difference in mean BMI between PRS deciles (reference is the first decile) in the Estonian Biobank (EstBB) target dataset for PRS derived from the multi-ancestry meta-analysis and UKBB. **D** BMI predictions of the multi-ancestry and UKBB-derived PRS in the EstBB target data

Given the improved fine-mapping resolution afforded by the multi-ancestry meta-analysis, we hypothesized that multi-ancestry PRS would have greater predictive power when applied in continental African populations from AWI-Gen than PRS derived from the European ancestry-specific UKBB. The multi-ancestry PRS was more strongly associated with BMI and explained a greater proportion of the trait variance in AWI-Gen (*p* = 8.31 × 10^−28^, 0.93%) than the UKBB PRS (*p* = 1.59 × 10^−16^, 0.54%) (Fig. 3, Table S1). Furthermore, the difference in mean BMI of individuals in the first decile compared to those in the tenth decile was more than two-fold greater for the multi-ancestry PRS (5.92 kg/m^2^) than for the UKBB PRS (2.86 kg/m^2^). To evaluate the generalizability of these findings to other ancestry groups, we repeated our analysis using BMI GWAS in 84,552 individuals of European ancestry in the Estonian Biobank (EstBB). We observed marginally greater BMI trait variance explained by the multi-ancestry PRS (*p* < 10^−300^, 6.72%) than the UKBB PRS (*p* < 10^−300^, 6.25%). These polygenic predictions in Europeans were seven-fold greater than those in continental Africans, indicating that the multi-ancestry PRS still needs a greater representation of continental Africans to enhance polygenic prediction. This is evidenced in the PRSCSx analysis, which showed a marked improvement in the prediction of BMI trait variance when the African populations (*p* = 9.47 × 10^−22^, 0.71% from *p* = 1.42 × 10^−14^, 0.45%) were added to the European and Asian discovery datasets in the development of the PRS as shown in Fig. 3B.

**Fig. 4:**
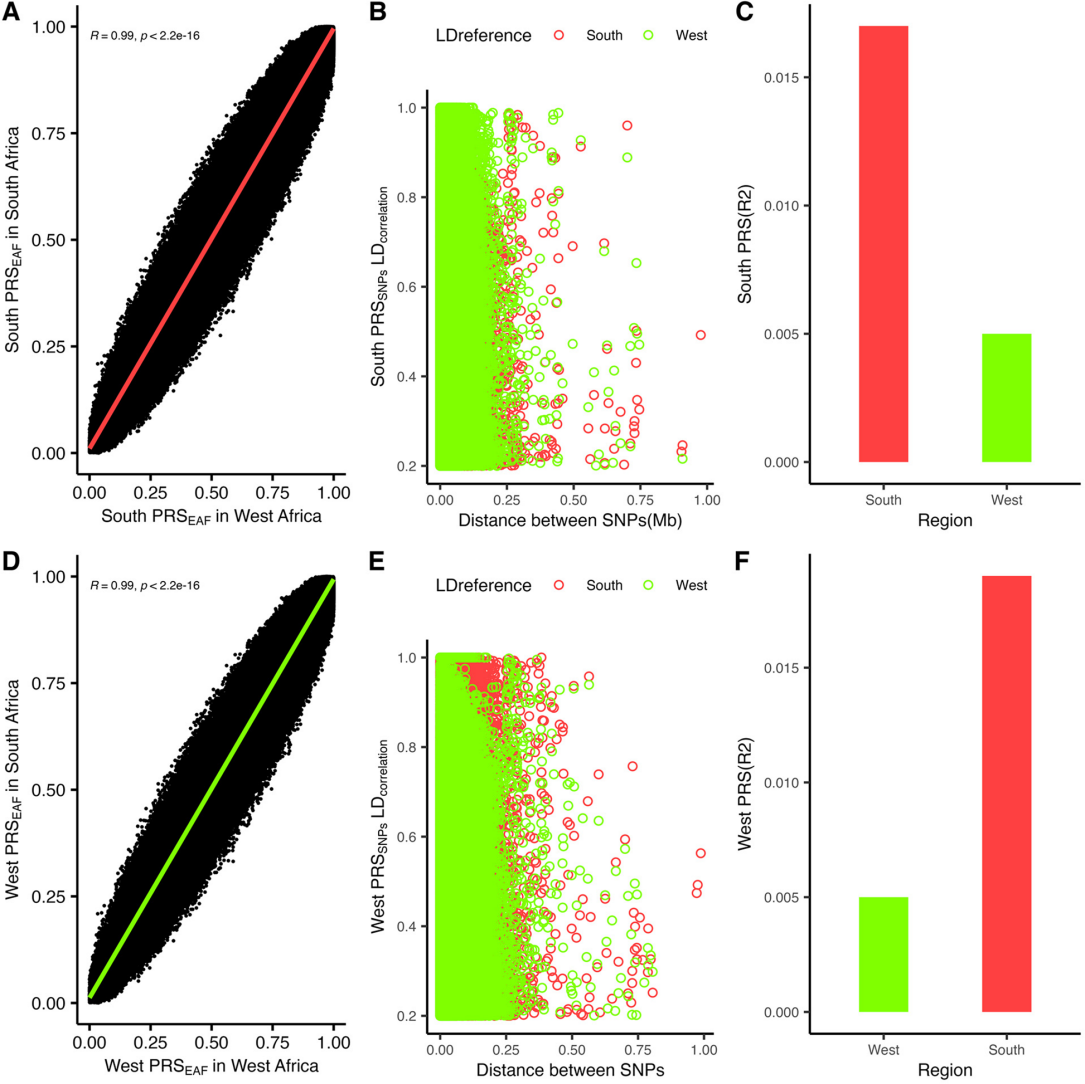
Genetic differences and region-specific PRS in regions of Africa. **A** Correlation of effect allele frequencies of the SNPs from the South Africa PRS in South and West Africa**. B** Extent of LD of SNPs from the South African PRS in South and West Africa. **C** BMI variance is explained by the South African PRS in South Africa and West Africa. **D** Correlation of effect allele frequencies of the West African PRS in South and West Africa. **E** Extent of LD of SNPs from the West African PRS in South and West Africa. **F** BMI variance is explained by the West African PRS in West Africa and South Africa

In view of the difference in the distribution of BMI between sexes in African populations (Fig. 4), we evaluated the predictive power of the multi-ancestry PRS in men and women from the AWI-Gen study. We observed a strong interaction of the PRS with sex (*p* = 1.4 × 10^−66^, Table S7), such that the difference in mean BMI between the first and tenth deciles was more than threefold greater in women (8.68 kg/m^2^) than in men (2.63 kg/m^2^). In sex-stratified analysis, the BMI trait variance explained by the multi-ancestry PRS was larger in women (1.09%, *p* = 3.8 × 10^−20^) than in men (0.86%, *p* = 4.8 × 10^−10^).

**Fig. 5:**
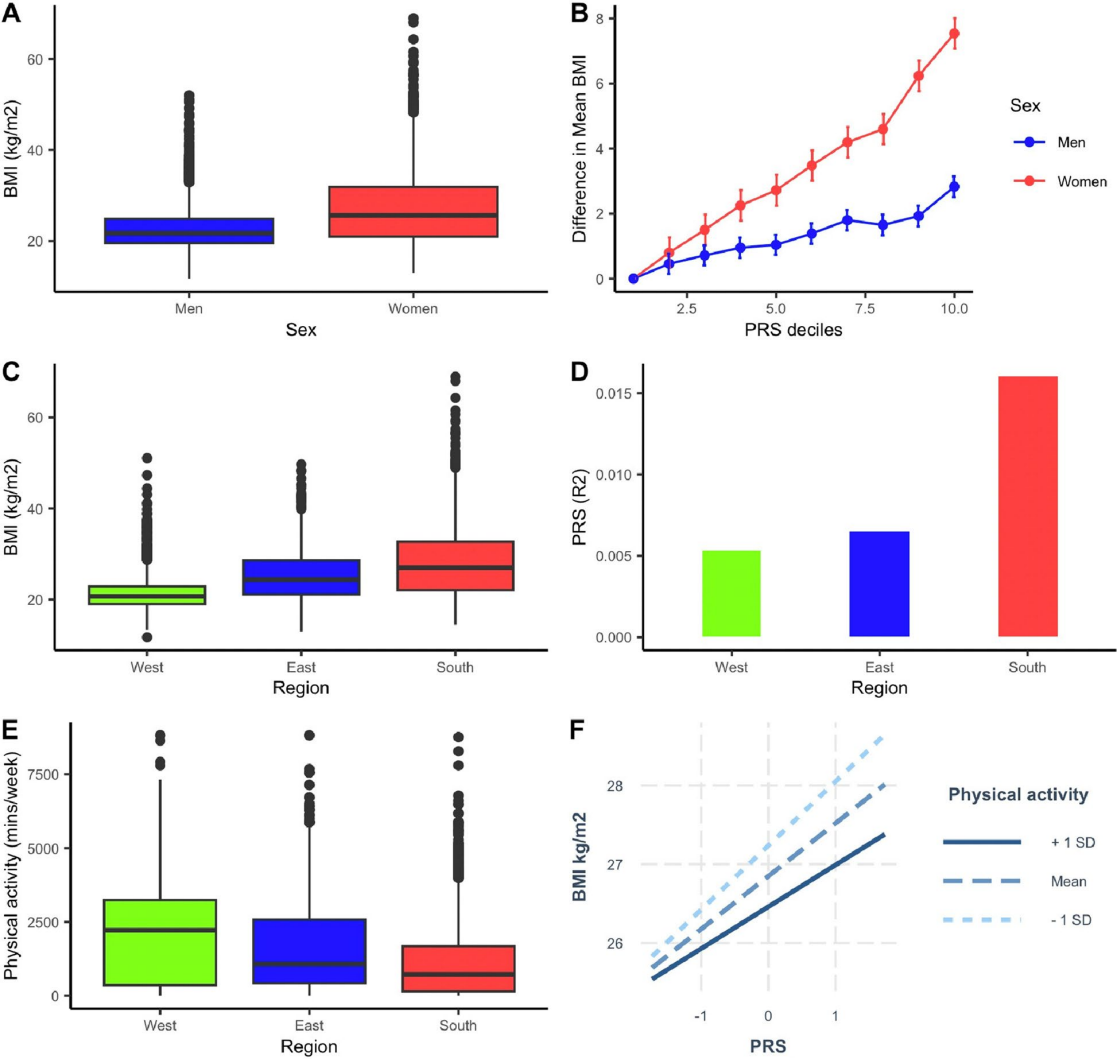
BMI distribution and PRS prediction in regions of Africa. **A** BMI distribution in men and women in the AWI-Gen study. **B** Difference in mean BMI of PRS deciles (reference is the first decile) in men and women in the target AWI-Gen dataset. **C** Distribution of BMI across West, East, and South regions of Africa. **D** Comparisons of the polygenic prediction of the multi-ancestry PRS in the West, East, and South regions of Africa. **E** Distribution of physical activity across the West, East, and South regions in Africa. **F** Interaction of the multi-ancestry PRS with physical activity in the AWI-Gen study

**Fig. 6:**
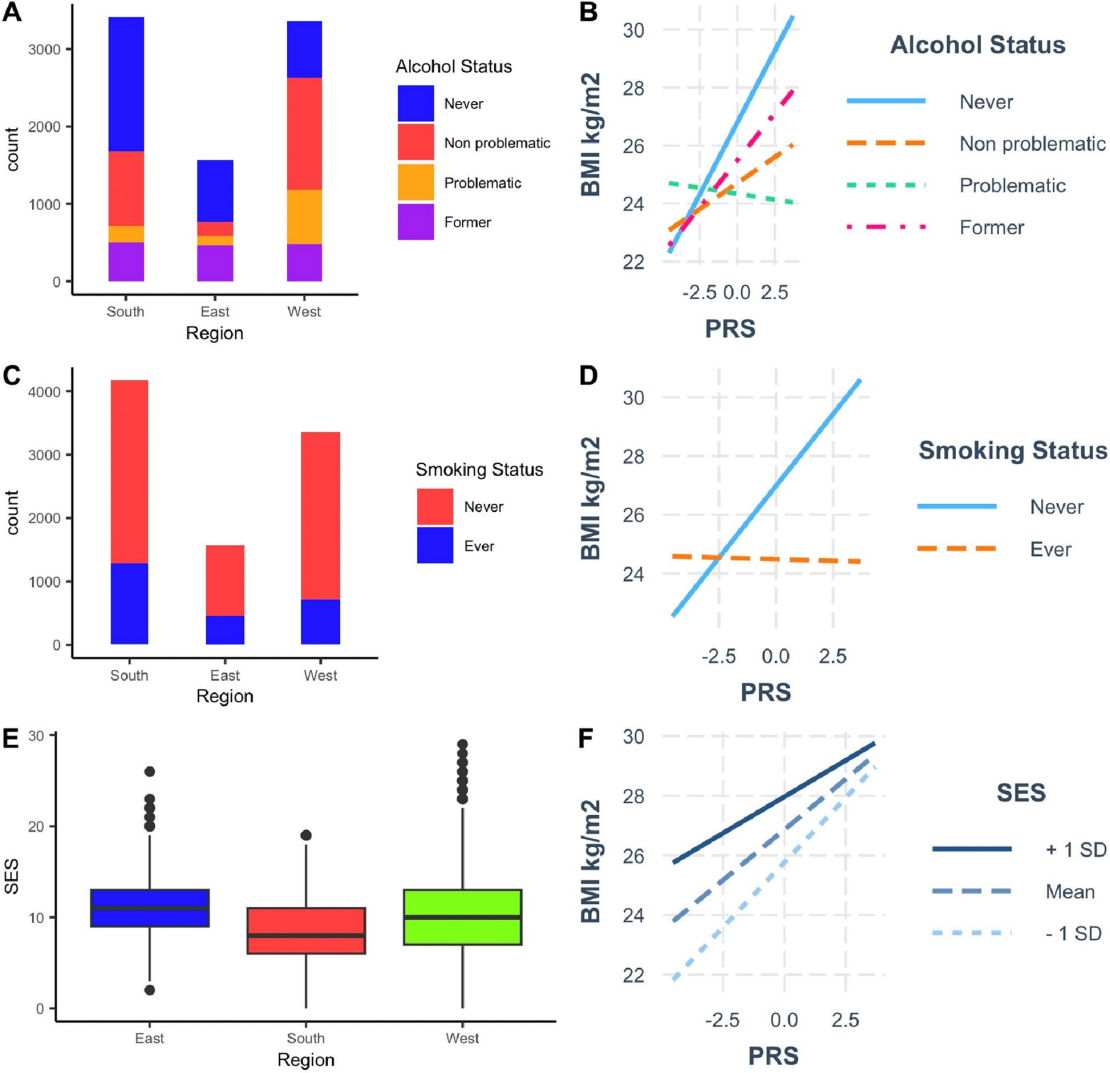
**A** Alcohol status and frequency (count) in regions of Africa in the AWI-Gen study. **B** Interaction of Alcohol status and multi-ancestry PRS in men and women in the target AWI-Gen dataset. **C** Smoking status and frequency (count) across West, East, and South regions of Africa. **D** Interaction of the multi-ancestry PRS with smoking status in the AWI-Gen study. **E** Distribution of socioeconomic status across the West, East, and South regions in Africa. **F** Interaction of the multi-ancestry PRS with socioeconomic status in the AWI-Gen study

Finally, given that AWI-Gen includes individuals from three regions of Africa, we investigated the performance of the multi-ancestry PRS across these different regions. We observed a significant interaction of the PRS with regions in Africa (East, West, and South) (*p* = 6.3 × 10^−7^) and noted that the PRS explained more BMI variance in South Africa (1.58%; 2.8 × 10^−21^) than West Africa (0.53%; 1.2 × 10^−5^). Such differences in performance could arise because of intra-region differences in genetic variation (allele frequency and LD patterns) and/or environment (lifestyle factors). Using the multi-ancestry meta-analysis, we first trained and validated region-specific PRS in West Africa and South Africa (Methods, Fig. 4, Table S2 and S3). For SNPs selected in each region-specific PRS, we observed a strong correlation in allele frequencies between West Africa and South Africa (*r* = 0.99, *p* < 2.2 × 10^−16^) and noted a similarity in the extent of LD in both regions. Regardless of the region where the PRS was developed, the PRS in South Africa explained greater BMI trait variance than in West Africa (Fig. 4), possibly due to interactions with lifestyle factors that vary between regions as shown in Figs. 5 and 6, Tables S7–S12. We observed a significant interaction of the multi-ancestry PRS with levels of physical activity (*p*_int_ = 0.018), socioeconomic status (*p*_int_ = 1.32 × 10^−3^), alcohol status (never vs problematic consumer) (*p*_int_ = 2.76 × 10^−8^), and smoking status (*p*_int_ = 1.64 × 10^−14^), which might be contributing to the variability in polygenic prediction in Africa.

## Discussion

We set out to understand the factors affecting the polygenic prediction of BMI and its variability across African regions. Our work shows that polygenic prediction is still low in African compared to European cohorts due to the limited representation of continental Africans in discovery GWAS. The multi-ancestry PRS was portable in Africa due to its enhancement of fine-mapping resolution. Sex differences in BMI distributions were also noted, with larger differences between the first and tenth decile in women compared to men. Gene-environment interactions were noted to have a larger effect on the generalizability of the PRS within the regions of Africa compared to allele frequencies and LD patterns of the SNPs in the PRS.

The PRS prediction of the multi-ancestry PRS was 7.2-fold less in a continental African cohort compared to a European cohort (0.93% vs 6.72% trait variance explained, respectively). Similarly, Martin et al. also reported a 4.5-fold difference in the prediction of the European PRS in Europeans vs African Americans [12]. Given that our multi-ancestry PRS comprised 70% Europeans, this might have contributed to the low predictivity in continental Africans. Adding the Africans enhanced prediction to the PRSCSx analysis. Though the prediction of the PRSCSx approach was lower compared to the multi-ancestry PRS as the prior is limited to HapMap SNPs which might not fully capture the genetic diversity in Africa. Thus, more representation of Africans in GWAS is required to enhance PRS prediction in Africa. Nonetheless, the multi-ancestry PRS enhanced the fine-mapping resolution, which enables the use of variants more causally associated with the trait in PRS development.

Notably, the differences in BMI between the first and last PRS deciles were more than threefold greater in women than in men. Thereby suggesting that the sex differences in BMI in Africans might be partly attributed to genetic factors. However, more studies evaluating the differences in heritability using sex-stratified GWAS in continental Africans are necessary. Nonetheless, future studies using deep phenotyping of the participants in the tails of the PRS distribution using a recall-by-genotype design can unravel the causal factors that may help explain the sex differences in BMI [22].

The generalizability of the multi-ancestry PRS within Africa was affected more by gene-environment interactions as opposed to the differences in allele frequencies and LD patterns. We noted an interaction of the PRS with lifestyle factors such as physical activity, socioeconomic status, smoking status, and alcohol status. Prior studies have reported similar interactions between the BMI PRS and socioeconomic status [23, 24] However, there are other lifestyle factors such as diet that we did not evaluate in this study which have been noted to interact with the PRS of BMI in Europeans [25]. Considering that lifestyle factors are challenging to measure objectively in resource-limited settings such as Africa. PRS tools that correct for variants that interact with these lifestyle factors need to be developed through approaches such as heterogeneity analysis of variance and identification of variance quantitative trait loci that are known to be candidate gene-interacting variants [26]. These approaches will need to include fine-mapping approaches that seek to ensure gene-environmental interactions with causal variants are not limited by gene-dependence bias [27]. Hierarchical symbolic regression approaches can be applied on these parameters to understand their contribution to the variability of PRS prediction [28]. This might help enhance the generalizability of the PRS in African populations.

## Conclusion

Our work demonstrates the improved transferability of multi-ancestry PRS over PRS derived from European ancestry GWAS for predicting BMI in populations from continental Africa. This may be driven by the refined localization of causal variants. Regional variability across Africa in polygenic prediction performance likely reflects genetic interactions with lifestyle factors that vary between populations, as we demonstrated that allele frequencies and LD patterns around associated variants were similar across African regions. Despite the improved performance of the multi-ancestry PRS, polygenic prediction of BMI in individuals from continental Africa remains low. The limited representation of continental African populations in genetic studies of complex human traits and diseases requires urgent attention to ensure Africans can benefit from precision medicine efforts.

### Supplementary Information

Additional file 1: Table S1. Development of the multi-ancestry (MAMA) and UKBB PRS in AWI-Gen and the Estonian Biobank. Figure S1. A. Grid of p-value thresholds (5E-08 to 1) at which PRS were computed to determine the best predictive one. B. Selected bar plots of p-value thresholds at which PRS were computed, indicating the best predictive one. Table S2. Development of the South African PRS using the AWI-Gen dataset. Table S3. Development of the West African PRS using the AWI-Gen dataset. Table S4. Clumping and LD parameters in the full AWIGen target dataset before splitting into training and validation. Table S5: Clumping and LD parameters in the AWIGen South target dataset before splitting into training and validation. Table S6. Clumping and LD parameters in the full AWIGen West target dataset before splitting into training and validation. Table S7. PRS and sex interaction models. Table S8. PRS and sex interaction models using inverse rank normalized BMI adjusted for age and principal components. Table S9. PRS and socioeconomic status interaction full model. Table S10. PRS and alcohol interaction full model. Table S11. PRS and smoking status full model. Table S12. PRS and physical activity interaction full model.

## Data Availability

The AWI-Gen dataset used in this study is available in the European Genome-phenome Archive (EGA) database (https://ega-archive.org/) under the study accession code EGAD00001006425 (https://ega-archive.org/datasets/EGAD00001006425). The genotype dataset accession code is EGAD00010001996 (https://ega-archive.org/datasets/EGAD00010001996) [17]. The availability of these datasets is subject to controlled access through, the Data and Biospecimen Access Committee of the H3Africa Consortium. The GWAS BMI summary statistics for APCDR can be accessed at(https://ftp.ebi.ac.uk/pub/databases/gwas/summary_statistics/GCST009001GCST010000/GCST009057/) [7]; PAGE at (https://ftp.ebi.ac.uk/pub/databases/gwas/summary_statistics/GCST008001GCST009000/GCST008025/) [8]; BBJ at (https://ftp.ebi.ac.uk/pub/databases/gwas/summary_statistics/GCST004001GCST005000/GCST004904/) [6] and UKBB (https://yanglab.westlake.edu.cn/data/ukb_fastgwa/imp/pheno/21001) [16]. The MAMA BMI PRS is available in the PGS catalog as score PGS004902.

## Abbreviations

BMI: Body mass index
GWAS: Genome-wide association study
PRS: Polygenic risk score
LD: Linkage disequilibrium
UK BB: United Kingdom Biobank
APCDR: Africa Partnership of Chronic Disease Research
BBJ: Biobank Japan
DDS: Durban Diabetes Study
DDC: Durban Diabetes Case–Control Study
PAGE: Population Architecture using Genomics and Epidemiology
MAF: Minor allele frequency
SNPs: Single nucleotide polymorphism
GPAQ: Global Physical Activity Questionnaire
LDSC: Linkage Disequilibrium Score Regression
EstBB: Estonian Biobank
